# Autophagy and senescence of rat retinal precursor cells under high glucose

**DOI:** 10.3389/fendo.2022.1047642

**Published:** 2023-01-04

**Authors:** Hanhan Peng, Wentao Han, Benteng Ma, Shirui Dai, Jianfeng Long, Shu Zhou, Haoyu Li, Baihua Chen

**Affiliations:** ^1^ Department of Ophthalmology, Second Xiangya Hospital, Central South University, Changsha, China; ^2^ Hunan Clinical Research Centre of Ophthalmic Disease, Changsha, China

**Keywords:** diabetic retinopathy (DR), autophagy, apoptosis, cellular senescence, differentially expressed genes, computational biology

## Abstract

**Backgrounds:**

Diabetic retinopathy (DR) is a common diabetic ocular disease characterized by retinal ganglion cell (RGC) changes. An abnormal environment, hyperglycemia, may progressively alter the structure and function of RGCs, which is a primary pathological feature of retinal neurodegeneration in DR. Accumulated studies confirmed autophagy and senescence play a vital role in DR; however, the underlying mechanisms need to be clarified.

**Methods:**

This study included the microarray expression profiling dataset GSE60436 from Gene Expression Omnibus (GEO) to conduct the bioinformatics analysis. The R software was used to identify autophagy-related genes (ARGs) that were differentially expressed in fibrovascular membranes (FVMs) and normal retinas. Co-expression and tissue-specific expression were elicited for the filtered genes. The genes were then analyzed by ontology (GO) enrichment analysis, Kyoto Encyclopedia of Genes and Genomes (KEGG) pathway enrichment analysis and Gene Set Enrichment Analysis (GSEA). R28 cells were cultured with high glucose, detected by reverse transcription-quantitative (RT-qPCR) and stained by apoptosis kit.

**Results:**

In the retina, 31 differentially expressed ARGs (24 up-regulated genes) were discovered and enriched. The enrichment results revealed that differentially expressed ARGs were significantly enriched in autophagy, apoptosis, aging, and neural function. Four hub genes (i.e., *TP53*, *CASP1*, *CCL2*, and *CASP1*) were significantly up-regulated. Upregulation of cellular autophagy and apoptosis level was detected in the hyperglycemia model *in vitro*.

**Conclusions:**

Our results provide evidence for the autophagy and cellular senescence mechanisms involved in retinal hyperglycemia injury, and the protective function of autophagy is limited. Further study may favour understanding the disease progression and neuroprotection of DR.

## Introduction

Diabetic retinopathy (DR) is a common microvascular complication of diabetes mellitus (DM), one of the world’s fastest-growing diseases that has become a leading cause of blindness in the working-age population (1). By 2040, 642 million people are expected to suffer from DM (2). Patients will face physical and mental suffering at an advanced stage. Within five years, almost half of patients with untreated proliferative diabetic retinopathy (PDR), the most resistant form of DR, will go blind (3, 4). A series of pathological changes are proved in PDR, including oxidative stress, endoplasmic reticulum stress, chronic retinal inflammation (5), retinal angiogenesis, retinal neuron injury (6), and other retinal disorders.

Retinal ganglion cells (RGCs) are bridges between photoreceptors and the brain to transmit visual information with the help of bipolar cells. The morphological and functional integrity of RGCs plays a vital role in maintaining normal retinal function. RGCs have their bodies in different retinal layers, making RGCs vulnerable to the retinal microenvironment and other cells (e.g., Müller cells, glial cells, astrocytes, and microglia). Excessive glucose in the retinal microenvironment (i.e., hyperglycemia) and reactive oxygen species (ROS) accumulation can alter the structure and function of RGCs, causing damage to them that progresses and leads to DR (7). RGCs injury is the onset of retinal neurodegeneration in DR. In advanced DR with proliferative fibrovascular membranes, retinal vascular occlusion, non-perfusion, retinal ischemia, and hypoxia can be observed clinically. These pathological changes are highly suggestive of elevated autophagy in retinal tissues (8, 9).

Autophagy refers to any intracellular process involving the lysosome’s degradation of cytosolic components. There are three distinct autophagic pathways: macroautophagy (autophagy), microautophagy and Chaperone-mediated autophagy. Autophagy is essential for survival, differentiation, development, and homeostasis and is widely involved in ocular diseases, such as DR and age-related macular degeneration (10–16).

Previous studies mainly focused on pericytes and RPE cells’ autophagy in DR. Wang et al. (17) found that the high expression of the autophagy-related gene (ARG) MAPK3 in retinal pigment epithelial cells might be involved in outer blood-retina barrier destruction in DR. Lin et al. (18) described in detail the effect of oxidative stress-activated autophagy on RGCs; however, it remains controversial whether the autophagy in RGCs is beneficial. It is worth noting that there is increasing evidence that autophagy is related to senescence, another common pathological change in endocrine and neurological diseases (19–21). Conclusively, the role of the double-edged sword of autophagy in DR is complicated; particularly significant is that the role and mechanism of RGCs autophagy in hyperglycemia and DR remain unclear.

This study investigated the potential mechanism for autophagy in DR *via in silico* and *in vitro* approaches. Our results showed that R28 cells under high glucose conditions exhibited significant upregulation of some ARGs, accompanied by significant apoptosis and senescence, consistent with the results of bioinformatics analysis.

## Materials and methods

### Microarray data and autophagy-related genes collection

The gene expression dataset GSE60436 was acquired from the Gene Expression Omnibus (GEO) database (http://www.ncbi.nlm.nih.gov/geo/) (22). Six fibrovascular membranes (FVMs) samples from PDR patients and three normal retinal tissue samples from donated eyes were included in this dataset. The original research summarized the raw gene expression data using the Illumina Bead Studio software. The thresholding of the signal values was normalized to the 75th percentile, and the baseline was transformed to the median using Genespring GX 11.0 software. The datasets were subjected to Welch’s t-tests and multidimensional false-discovery control (the cutoff value between FVMs and retina was 0.5%).

In this study, the expression data were normalized using the ‘limma’ package in R software (4.2.0 GUI). The 222 ARGs were obtained from The Human Autophagy Database (http://www.autophagy.lu/index.html), which provides a comprehensive and up-to-date list of human genes and proteins involved directly or indirectly in autophagy as described in the literature.

### Differential expression analysis of autophagy-related genes

Using the ‘limma’ package to screen the DEGs, *P* value 0.05 and |log2FoldChange| > 1 filters were used to narrow the results. R software was used to visualize the DEGs and autophagy-related DEGs. The expression in the retina of differentially expressed ARGs was evaluated and visualized using BioGPS website (http://biogps.org) (23–25) and GraphpadPrism software (version 9.0.1 for macOS). Expression values from Affymetrix chips relate to fluorescence intensity, which was mostly summarized using various data processing algorithms, gcrma, in BioGPS.

### Construction of co-expression network

The Search Tool for the Retrieval of Interacting Genes/Proteins (STRING v.11.5, https://string-db.org/) database and Cytoscape (v.3.8.0) were used to establish and perform the visualization of the protein-protein interaction (PPI) network. Ten hub genes were identified by the cytoHubba plugin in Cytoscape with the Maximal Clique Centrality (MCC) method. The interactions between the differentially expressed ARGs were analyzed by GeneMANIA (http://genemania.org/) (26), a gene function prediction tool for the genetic interaction (GI) network. Additionally, the bulk tissue expression of the top ten hub genes was analyzed on GTEx Portal (https://www.gtexportal.org/) (27, 28).

### Gene ontology and kyoto encyclopedia of genes and genomes pathway enrichment

Based on the differentially expressed ARGs, gene ontology (GO) and Kyoto Encyclopedia of Genes and Genomes (KEGG) pathway enrichment were performed using the Database for Annotation, Visualization and Integrated Discovery (DAVID) v2022q2 (https://david.ncifcrf.gov/) (29, 30). The GO analysis contained biological processes (BPs), cellular components (CCs), and molecular functions (MFs).

### Gene set enrichment analysis for pathways

Gene Set Enrichment Analysis (GSEA) was used to investigate the probable biological functions and pathways involved in DR in FVMs and normal retinas. GSEA was carried out using WebGestalt (WEB-based Gene SeT AnaLysis Toolkit, http://www.webgestalt.org/) (31–34). The top ten significant terms were shown.

### Cell line and culture

Rat retinal precursor R28 cells were cultured in Dulbeccos Modified Eagle Medium (DMEM, BasalMedia, China) containing 10% fetal bovine serum (FBS, Gibco, USA) and 1% Antibiotic-Antimycotic (Gibco, USA) at 37°C with 5% CO_2_. Cells were rinsed with PBS (Gibco, USA) and treated with 0.05% trypsin (Gibco, USA) for passage at 1: 3.

In subsequent experiments, the logarithmic growth phase cells were fed with a medium containing either 30 mM D-glucose (HG group) or a medium containing 5.6 mM D-glucose (LG group) for 48 hours, as appropriate.

### RNA extraction and reverse transcription-quantitative PCR

Cells were placed in TRIzol (TaKaRa Biotechnology, China) reagent for RNA isolation. Chloroform (Macklin Reagent, Beijing, China) was applied to the cells for 15 seconds before being incubated at room temperature for 10 minutes. The top layer was transferred to a fresh microcentrifuge tube and mixed with isopropanol after being centrifuged at 10,000 × g for 10 minutes at 4°C. The material was centrifuged, rinsed, and then RNase-free water was used to elute the total RNA. Using a NanoVue Plus spectrophotometer (GE Healthcare, USA), the size and purity of the RNA were evaluated for quality. The extracted total RNA was treated to reverse transcription using a RevertAid Master Mix kit to convert isolated RNA into cDNA (Thermo Scientific, USA).

On a StepOne Plus Real-Time PCR System (Applied Biosystems, USA), cDNA from the cells was utilized as the template for reverse transcription-quantitative PCR (RT-qPCR) amplification using TB Green Premix Ex TaqII (TaKaRa Biotechnology, China). In separate experiments, RT-qPCR was performed to assess the transcript levels of *TP53*, *CASP3*, *CCL2*, *HIF1A*, *CASP1*, *CDKN1A*, *CDKN2A*, and *β-actin* in cell samples using the gene-specific primers shown in **Supplementary Table S1**. Target gene expression levels were calculated by 2^-△△^ cycle threshold (Ct) method and normalized to that of *β-actin*.

### Flow cytometry

As reported previously, apoptosis was evaluated by flow cytometry (35). Briefly, R28 cells were harvested with EDTA-free trypsin (Gibco, USA) and centrifuged at 300 × g for 5 minutes at 4 °C. The cells were washed with pre-cooled PBS twice and then stained with Annexin V-FITC Apoptosis Detection Kit (Vazyme, China). Each sample was resuspended with 100 μL Binding Buffer and stained with 5 μL Annexin V-FITC and 5 μL PI Staining Solution at dark at room temperature for 10 minutes. After adding 400 μL Binding Buffer, apoptotic cells were detected using a FACS Dxp AthenaTM (Cytek, Fremont, USA) and analyzed using FlowJo v10.0 (FlowJo Software, USA).

### Statistical analysis

Statistical analyses were carried out by GraphpadPrism software. The statistical significance for RT-qPCR and flow cytometry were compared using Two-way and One-way ANOVA, respectively. The difference was statistically significant when *P* < 0.05.

## Results

### Data collection and differentially expressed autophagy-related genes identification

**Figure 1:**
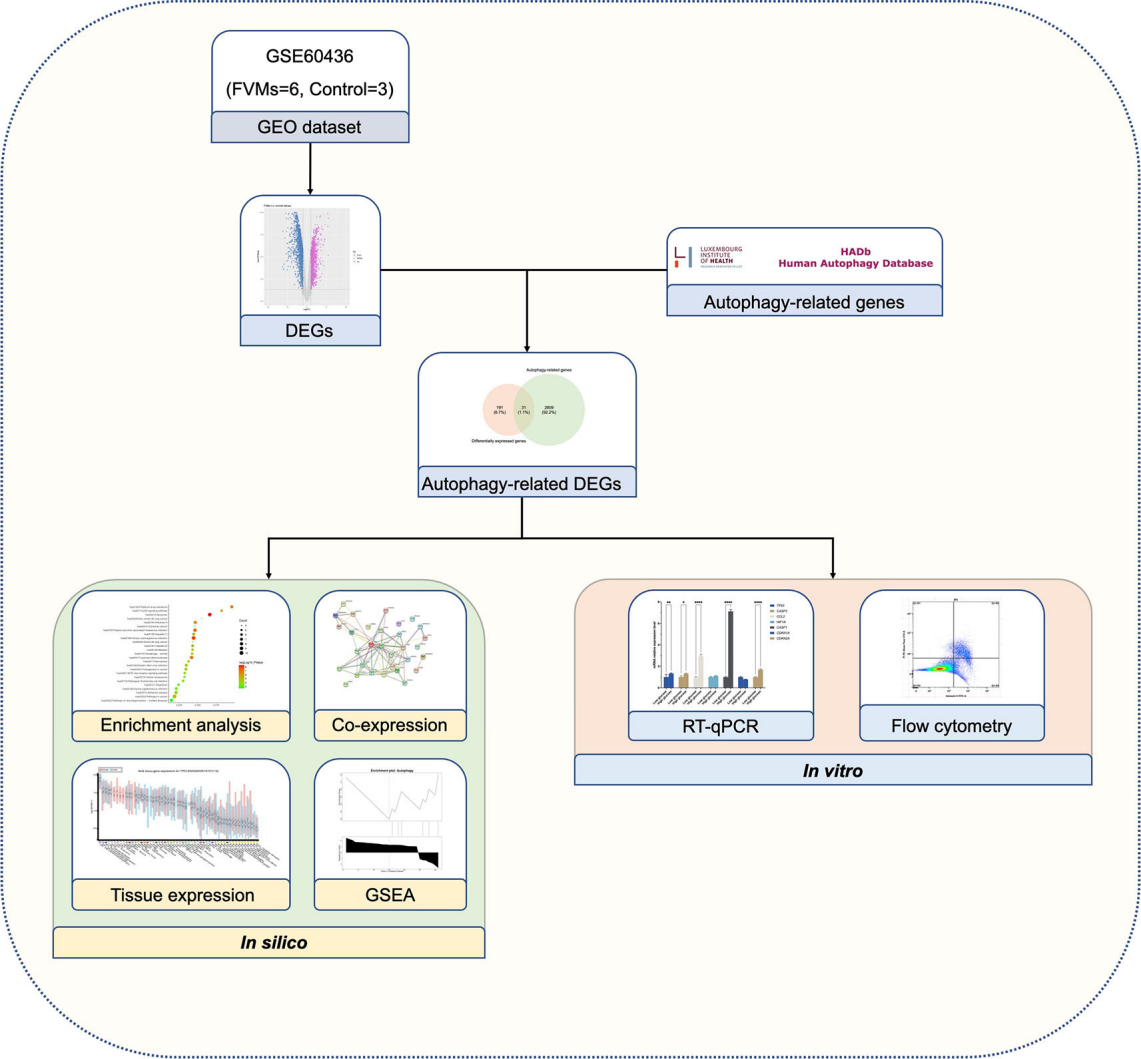
Schematic overview of the study design.

**Figure 2:**
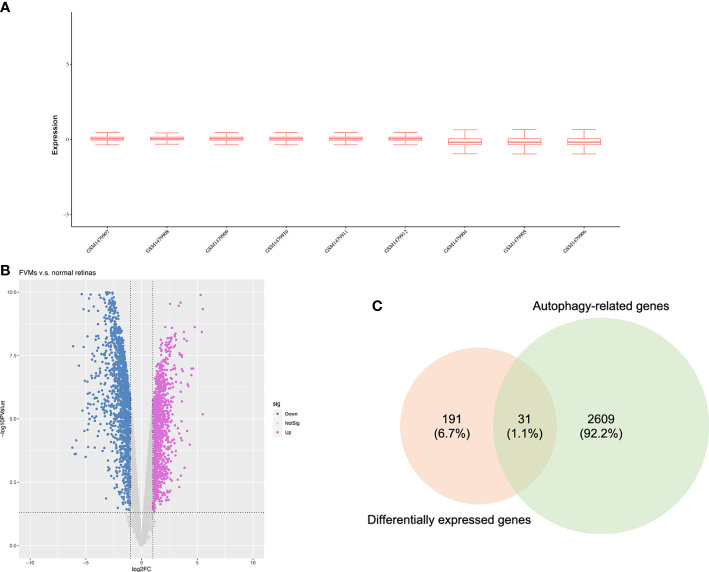
Identification of differentially expressed autophagy-related genes. **(A)** Box plots for the expression profiles after normalization. **(B)** Volcano plot of differentially expressed genes between the fibrovascular membranes and normal retinas. **(C)** Venn diagram of overlapped genes between differentially expressed genes and autophagy-related genes.

**Figure 3:**
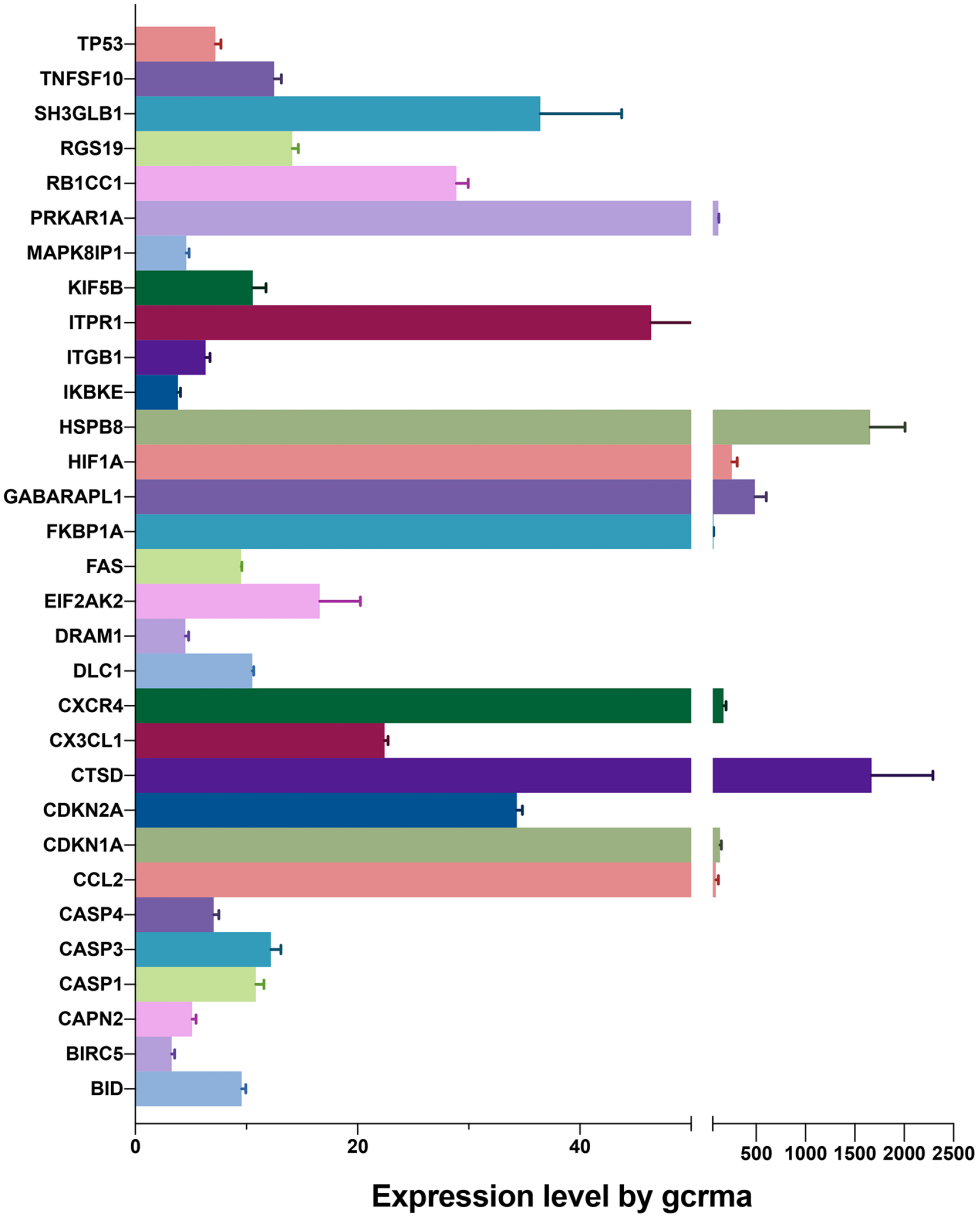
Expression levels of differentially expressed genes identified by BioGPS in retinal tissues.

As shown in **Figure 1**, we firstly downloaded the expression profiling by array dataset GSE60436 (GPL6884, Illumina HumanWG-6 v3.0 expression BeadChip) from the GEO database. This dataset included six FVMs (three active FVMs: age 57.3 ± 2.1 years, one man and two women, duration of diabetes 15.7 ± 2.1 years, glycosylated hemoglobin 8.3%; three inactive FVMs: age 48.0 ± 13.0 years, one man and two women, duration of diabetes 12.3 ± 2.5 years, glycosylated hemoglobin 6.1%) and three normal retinas as control. After normalizing the expression data with R (**Figure 2A**), we found 1,442 up-regulated genes and 1,618 down-regulated genes in FVMs compared to control samples (**Figure 2B**). Among these DEGs, 31 ARGs were screened (**Figure 2C**), seven were down-regulated, and 24 were up-regulated (**Table 1**). The down-regulated autophagy-related DEGs were *GABARAPL1*, *PRKAR1A*, *HSPB8*, *CTSD*, *MAPK8IP1*, *ITPR1*, and *CX3CL1*. The top ten DEGs with the most significant upregulation were *CDKN2A*, *CDKN1A*, *ITGB1*, *DRAM1*, *CASP1*, *CCL2*, *CASP4*, *BIRC5*, *CXCR4*, and *CASP3*. To gain further insight into the tissue distribution of genes of interest, we searched the BioGPS database. The results indicated that the autophagy-related DEGs were enriched in human retina tissue, particularly *CCL2*, *CDKN1A*, *CTSD*, *HIF1A*, *HSPB8* and other genes (**Figure 3**).

**Table 1:** The differentially expressed autophagy-related genes in disease samples compared to control samples.

Genes	Changes	logFC	*P*-value	Adjusted *P*-value	Probe ID
*GABARAPL1*	Down	-2.5881215	3.92E-12	1.07E-09	ILMN_2151281
*PRKAR1A*	Down	-2.1424335	2.11E-02	3.23E-02	ILMN_2389590
*HSPB8*	Down	-1.7843169	1.84E-05	2.53E-04	ILMN_1791280
*CTSD*	Down	-1.6241016	1.07E-04	1.06E-03	ILMN_1674038
*MAPK8IP1*	Down	-1.3304423	1.74E-08	8.65E-07	ILMN_1680434
*ITPR1*	Down	-1.2858511	5.64E-07	1.47E-05	ILMN_1789505
*CX3CL1*	Down	-1.0935473	9.72E-06	1.48E-04	ILMN_1654072
*CAPN2*	Up	1.0260874	7.42E-06	1.19E-04	ILMN_1716057
***TP53***	Up	1.0552317	2.14E-06	4.30E-05	ILMN_1779356
*EIF2AK2*	Up	1.0635111	8.36E-05	8.64E-04	ILMN_1706502
***TNFSF10***	Up	1.0813628	6.97E-06	1.13E-04	ILMN_1801307
***FAS***	Up	1.1846158	5.54E-08	2.21E-06	ILMN_1808132
*RB1CC1*	Up	1.252969	8.88E-08	3.24E-06	ILMN_1736796
*SH3GLB1*	Up	1.2836526	2.97E-06	5.62E-05	ILMN_1766045
*IKBKE*	Up	1.2988779	1.31E-07	4.47E-06	ILMN_1755024
***HIF1A***	Up	1.2994709	1.21E-04	1.16E-03	ILMN_1763260
*RGS19*	Up	1.2997582	4.04E-09	2.71E-07	ILMN_1677085
*DLC1*	Up	1.3426179	8.67E-05	8.89E-04	ILMN_1698020
*KIF5B*	Up	1.3494477	2.36E-06	4.66E-05	ILMN_1788160
*BID*	Up	1.4363423	4.76E-08	1.97E-06	ILMN_1763386
*FKBP1A*	Up	1.5002887	7.00E-06	1.13E-04	ILMN_1683658
***CASP3***	Up	1.5355761	1.24E-07	4.27E-06	ILMN_2388155
***CXCR4***	Up	1.5825074	3.24E-07	9.38E-06	ILMN_2320888
*BIRC5*	Up	1.6013535	2.21E-05	2.93E-04	ILMN_2349459
*CASP4*	Up	1.6702278	1.18E-10	1.59E-08	ILMN_1678454
***CCL2***	Up	1.7023316	1.03E-08	5.67E-07	ILMN_1720048
***CASP1***	Up	1.7429502	5.34E-09	3.40E-07	ILMN_2326509
*DRAM1*	Up	1.7657773	9.13E-09	5.15E-07	ILMN_1669376
***ITGB1***	Up	2.1046631	2.60E-07	7.90E-06	ILMN_1714820
*CDKN1A*	Up	2.2767255	9.87E-11	1.40E-08	ILMN_1784602
***CDKN2A***	Up	2.4632342	1.31E-06	2.90E-05	ILMN_1717714

The top ten hub genes are shown in bold.

### Co-expression network construction and hub genes identification

**Figure 4:**
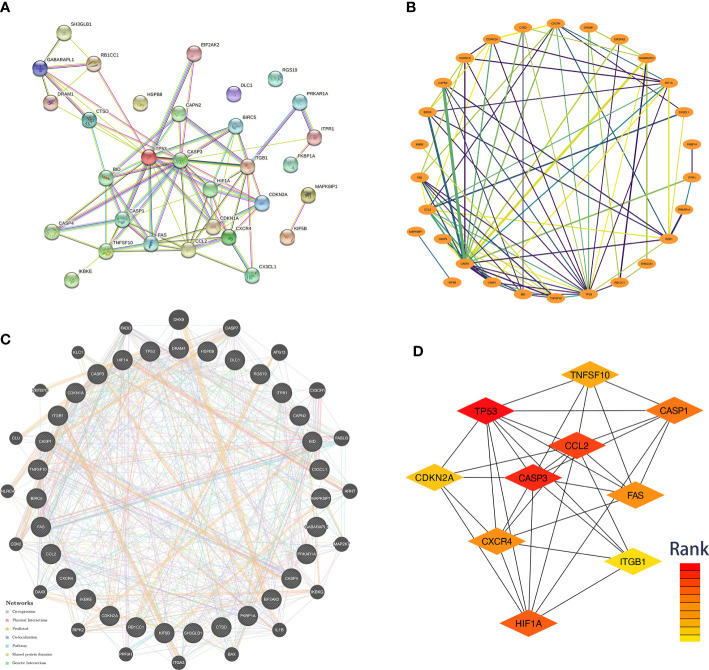
Co-expression network and identification of hub genes. **(A)** The PPI network between the differentially expressed autophagy-related proteins was established by using the STRING database. The node represents the protein, and the edge represents the relationship between the proteins. **(B)** The PPI network between the differentially expressed autophagy-related proteins was established by using Cytoscape with data from the STRING database. The edge width represents edge betweenness. The edge color represents combined score. **(C)** The top ten hub genes were screened by cytoHubba plugin. Nodes from red to yellow indicate descending rank. **(D)** The genetic interaction network between the differentially expressed autophagy-related proteins was established by using the GeneMANIA. PPI, protein-protein interaction.

**Figure 5:**
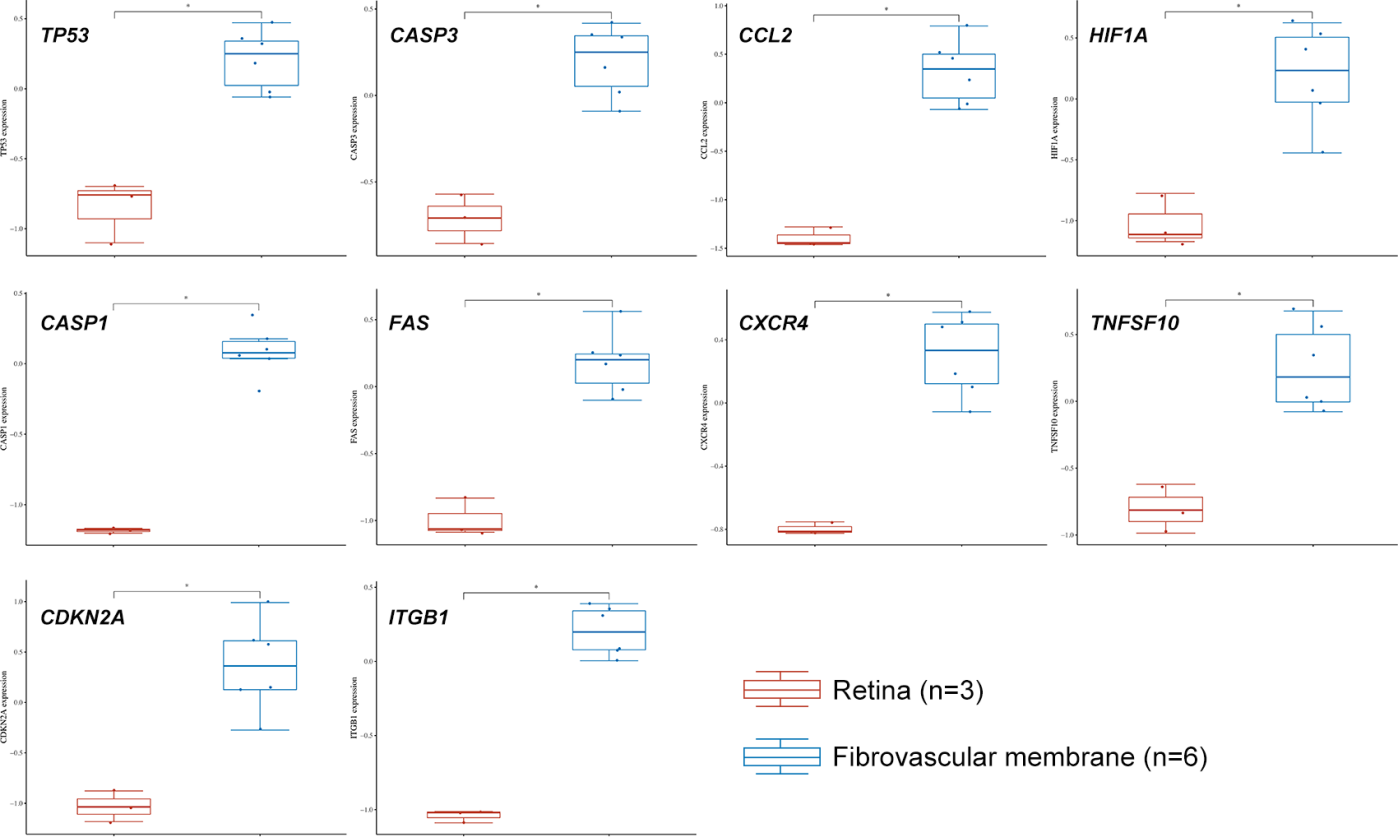
The expression distribution of *TP53*, *CASP3*, *CCL2*, *HIF1A*, *CASP1*, *FAS*, *CXCR4*, *TNFSF10*, *CDKN2A*, and *ITGB1* in normal retina and fibrovascular membrane. The statistical difference of two groups was compared through the Wilcox test. *P < 0.05.

We used data from STRING and GeneMANIA to establish PPI and GI networks for investigating the interactions at the protein and gene levels. We used the MCC method to obtain the hub genes based on all the autophagy-related DEGs. These genes have extensive interactions at the protein (**Figures 4A, B**) and gene levels (**Figure 4C**). The top ten hub genes with descending rank were *TP53*, *CASP3*, *CCL2*, *HIF1A*, *CASP1*, *FAS*, *CXCR4*, *TNFSF10*, *CDKN2A*, and *ITGB1* (**Figure 4D**). All these ten genes were significantly up-regulated in FVMs, which indicated autophagy might be overactivated in DR (**Figure 5**). To deeper understand the bulk tissue expression of the top ten hub genes, the GTEx Portal was accessed (**Supplementary Figure S1**).

### Functional and pathway enrichment

**Figure 6:**
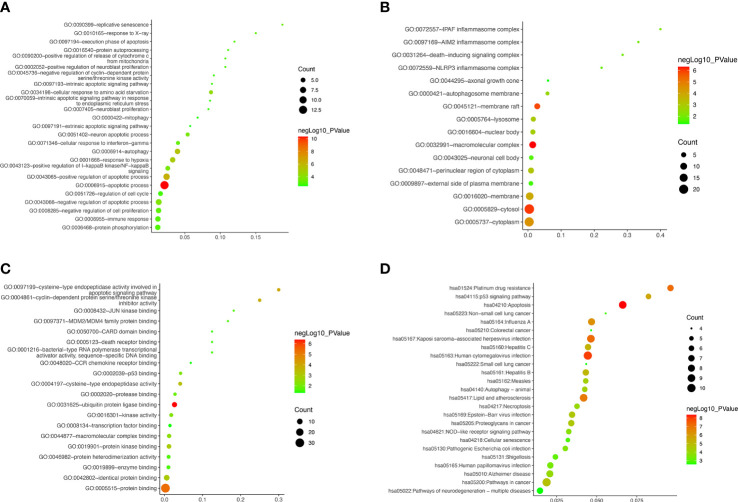
Gene ontology and Kyoto Encyclopedia of Genes and Genomes Pathway Enrichment of differentially expressed autophagy-related genes. **(A–D) ** Bubble plot for biological processes, cellular components, molecular functions, and enriched pathways, respectively.

The GO, KEGG and GSEA enrichment was carried out to study the functions, subcellular localizations and pathways of the autophagy-related DEGs. For BPs, the DEGs were mainly enriched at autophagy, apoptosis, mitophagy, immune response, and other terms (**Figure 6A**). For CCs, in addition to inflammasomes and autophagosomes, DEGs were enriched in the neuronal cell body, axonal growth cone, nuclear body, and cellular membranes (**Figure 6B**). MFs revealed the DEGs involved in the death receptor binding and binding of p53 and other proteins and enzymes (**Figure 6C**). Furthermore, autophagy, apoptosis, cellular senescence, necroptosis, and neurodegeneration pathway were popular KEGG terms for DEGs (**Figure 6D**). The autophagy, apoptosis, cellular senescence, and p53 signaling were also confirmed by GSEA pathway enrichment (**Supplementary Figure S2**). To further validate the apoptosis level, we performed flow cytometry. The results showed that the apoptosis level of R28 cells was significantly increased after high glucose challenge (**Supplementary Figure S3**). Compared with the control group, the levels of late apoptosis and total apoptosis were significantly increased in the high glucose stimulation group (*P* < 0.01). Although not statistically different, the HG also had higher levels of early apoptosis than the LG.

Moreover, the down-regulated genes were primarily enriched in neuron protection, cell adhesion, and metabolism terms (**Supplementary Table S2**). In this study, most autophagy-related DEGs were up-regulated and associated with angiogenesis, inflammation, fibrosis, senescence, and programmed cell death (**Supplementary Table S3**). Cellular senescence and cell death (i.e., autophagy, apoptosis, and necroptosis) were speculated to get involved in DR occurrence and development. It is worth noting that some neuronal terms were enriched, which suggested that retinal nerve damage is also one of the main pathological changes of DR.

### Differentially expressed autophagy-related genes expression validation

**Figure 7:**
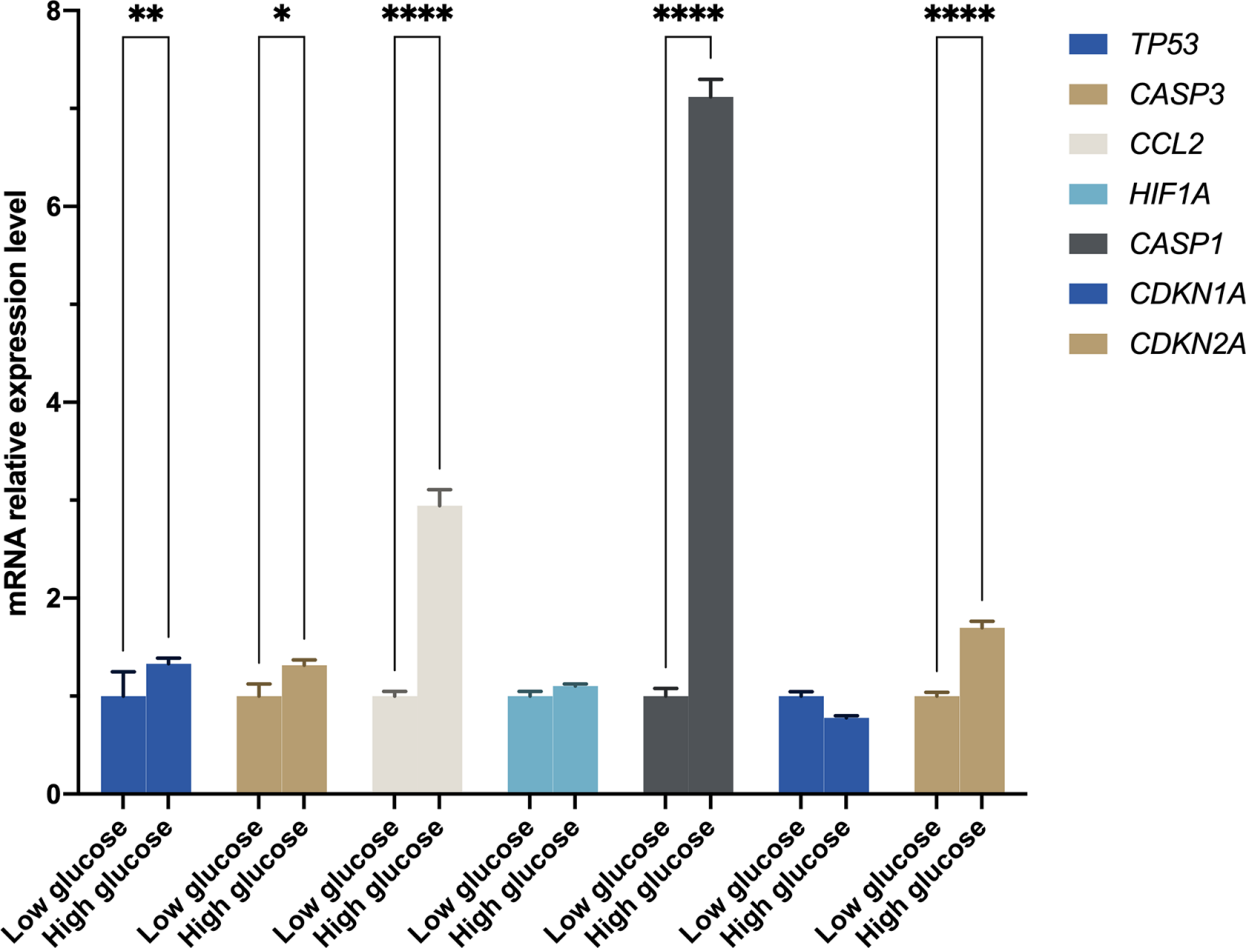
The mRNA level was evaluated in R28 cells by quantitative real-time polymerase chain reaction. *P*-values were calculated using Two-way ANOVA. *P < 0.05; **P < 0.01; ****P < 0.0001.

It was found that RGCs autophagy is highly involved in DR development. Our results mentioned above suggested autophagy was up-regulated in DR samples. In this present study, we incubated R28 cells with high glucose (30 mM) for 48 hours to mimic the retinal hyperglycemic environment and measured hub gene expression by RT-qPCR. The results were generally consistent with expectations that *TP53*, *CASP3*, *CCL2*, and *CASP1* mRNA were significantly higher in the HG group than in the LG group (**Figure 7**). However, *HIF1A* remains unchanged statistically, although a slight uptrend was observed. In addition to the top five hub genes, we detected two senescence-associated genes, *CDKN1A* and *CDKN2A*, in expression. Interestingly, *CDKN2A*, rather than *CDKN1A*, was significantly up-regulated in the HG group (**Figure 7**).

## Discussion

In this study, we investigated the RGCs autophagy in DR. DR is a complex and gradual chronic complication of diabetic patients. Previous studies have shown several metabolic pathways involved in DR pathological changes. It is worth noting that these pathways’ abnormal signaling may lead to microvascular complications (e.g., blood-retinal barrier destruction, microvascular leakage, and edema) and neuron disorders (e.g., neurodegeneration and neuroinflammation). The neurovascular unit (NVU), which is made up of neurons, vascular tissue, and supporting glial cells, is found in the retina (36). The ocular NVU may be affected if the blood-retinal barrier (BRB) breaks down. DR has traditionally been associated with retinal angiopathy or vasculopathy, however there is evidence of neurological impairments (37). As a result, diabetic retinal neurodegeneration (DRN) and diabetic retinal vasculopathy (DRV) are currently thought to be two connected parts of DR. The BRB breakdown and retinal vascular disorders in DR are widely studied. Notably, Wang et al. (17) have primarily demonstrated the role of RPE autophagy in BRB damage in DR. DRN is now considered an initial event contributing to DRV and is detectable before the clinical appearance of DRV (38). In-depth exploration of DRN is more helpful to directly understand the mechanism and prevention methods of blindness caused by DR.

In this study, we focused on RGCs loss, one of the most significant features of DRN. We screened a GEO dataset with an autophagy gene list from The Human Autophagy Database and found that 31 ARGs were significantly differentially expressed. The top ten hub genes were screened out *via* the MCC method (*TP53*, *CASP3*, *CCL2*, *HIF1A*, *CASP1*, *FAS*, *CXCR4*, *TNFSF10*, *CDKN2A*, and *ITGB1*). Additionally, these genes were highly enriched in retinal tissue, which suggested that retinal autophagy levels might be altered in DR. Further analyses showed that the up-regulated ARGs were associated with autophagy, apoptosis, cellular senescence, necroptosis, and neurodegeneration. Our results were consistent with clinical findings that long-term and chronic hyperglycemia may cause a significant and extensive reduction of RGCs and thinning of the RGCs layer in DM (39). These preliminary results confirmed the theory of nerve damage in DR.

To further verify the genes of interest expression in RGCs, we construct an *in vitro* high glucose model using R28 cells to mimic the retina environment of DR. The RT-qPCR results of the top five hub genes and two senescence-related genes (i.e., *CDKN1A* and *CDKN2A*) showed that *TP53*, *CASP3*, *CCL2*, *CASP1*, and *CDKN2A* were significantly up-regulated in HG than LG (**Figure 7**). In response to various cellular stressors, the *tumor protein p53* (*TP53*) controls the expression of target genes, causing cell cycle arrest, apoptosis, autophagy, senescence, DNA repair, and adjustments in cellular metabolism (40). Previous studies found higher *TP53* expression in diabetic retinal tissue than in control samples and considered *TP53* polymorphism a genetic marker for diabetic complications (41–43). The caspase family has been proven to play a vital role in DR, particularly *CASP3* and *CASP1*. *CASP3* and *CASP9* showed increased expression in RGCs, suggesting early apoptosis of RGCs (44). In addition to RGCs, animal studies showed that increased *CASP3* could reduce the rod outer segments length, increase early apoptosis in photoreceptors in DM, and increase apoptosis of dopaminergic amacrine cells in the retina with hyperglycemia (45–47). The direct activation of *CASP1* participated in signaling transformation in the canonical pathway of pyroptosis. Wang et al. (48) have confirmed that pyroptosis and inflammation improve DR’s occurrence and development. *CCL2* gets involved in neuroinflammation. Treatment with CCL2 could promote macrophage-like cells recruited on the retina surface, a characteristic of vision-threatening retinal vascular diseases like DR and retinal vein occlusion (49). The regulation between *HIF1A* and *TP53* is bidirectional and complex, and one of the possible reasons there was no statistical difference in *HIF1A* expression is that it was suppressed by highly expressed *TP53* (50). The *CDKN2A* was highly expressed in HG rather than LG; however, no significant difference was observed for *CDKN1A*. This result indicated that the R28 cells in HG suffered cellular senescence, and cells have entered the senescence maintenance stage under a long-term high-glucose environment.

Apoptosis is another meaningful programmed cell death and shares some regulatory genes with autophagy. Our results showed a significant increase in apoptotic cells in the HG group. Appropriate apoptosis and autophagy are protective mechanisms of cells to scavenge unhealthy cells and metabolic wastes to avoid carcinogenesis and aging. Interestingly, as we showed, excessive activation of apoptosis and autophagy under long-term chronic hyperglycemia did not alleviate aging, but further aggravated RGCs loss. Additionally, the period of autophagy’s protective effect might be shortened with acute injury (51). Although inhibition of autophagy decreased the apoptosis of RGCs in diabetic retinas (9), in high glucose, the self-rescue effect of RGCs *via* autophagy is limited. Therefore, harnessing the protective effects of apoptosis and autophagy in short- and long-term hyperglycemic environments in DR is a complex topic that requires more exploration.

We acknowledge the limitations that this study used RGCs to verify the differences between FVMs and normal retinal tissue. Due to the difficulty of obtaining human retinal tissue, there are few sequencing results on the retina and RGCs. By mining the differentially expressed genes in FVMs and normal retinas, we obtained genes potentially altered in retinal under a high glucose environment and inferred the damage targets of high glucose to RGCs. Like the studies by Wang et al. (17, 48), we extrapolated the conclusions of the initial results to provide a new perspective on the pathogenesis of DR. This study preliminarily confirmed that autophagy and aging of RGCs are involved in high glucose injury combined with sequencing results and gene expression verification. Although R28 cells display both glial and neuronal cell markers, they are widely used in studies. They can respond to various stimuli with advantages including unlimited lifespan, not being tumorigenic *in vivo*, and closely simulating a retinal explant (52).

## Conclusion

In summary, we investigated the potential mechanism of autophagy and senescence in DR *via* bioinformatics approaches and validation *in vivo*. We found that under high glucose conditions, the levels of some autophagy- and senescence-related genes in R28 cells were significantly increased. These results initially revealed that autophagy and senescence-related genes (e.g., *TP53* and *CDKN2A*) regulated retinal cells involved in DR pathogenesis and challenged the protective effect of enhanced autophagy in retinal cells under high glucose environment. Further studies could provide novel mechanistic insight into DR.

## Data availability statement

The original contributions presented in the study are included in the article/**Supplementary Material**. Further inquiries can be directed to the corresponding authors.

## Author contributions

Conceptualization: HL and BC. Methodology and software: HP, WH, and BM. Visualization: WH and BM. Formal analysis and data curation: HP, WH, BM, and SD. Investigation and validation: BM, SD, JL, and SZ. Writing: HP and WH. Review and editing: HP, HL, and BC. Supervision and acquisition of funding: HL and BC. All authors contributed to the article and approved the submitted version.
