# Supplementary figures and images for: Autophagy and senescence of rat retinal precursor cells under high glucose
Source: Front Endocrinol (Lausanne). 2023 Jan 4;13:1047642. doi: 10.3389/fendo.2022.1047642 (PMC9846177; doi:10.3389/fendo.2022.1047642)

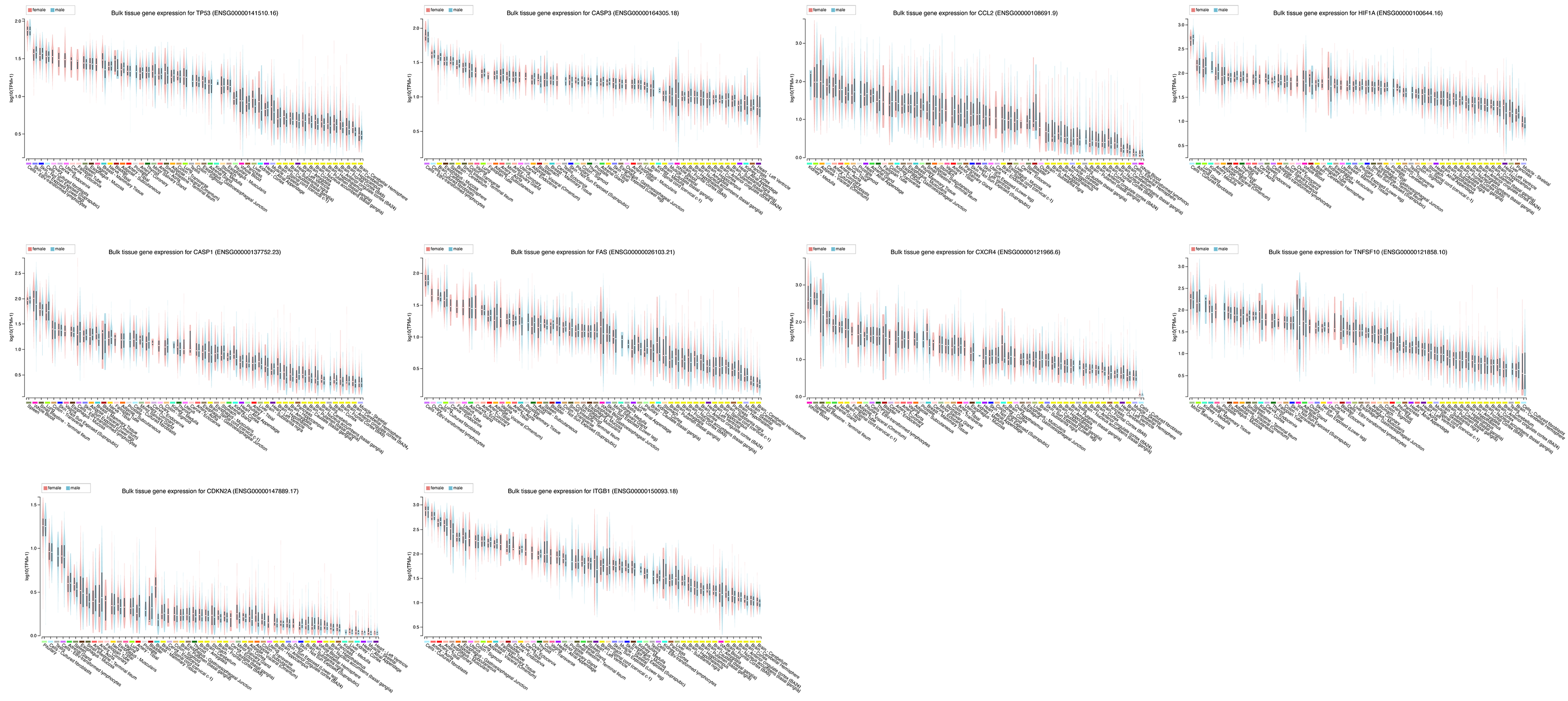

Supplement: Supplementary file 1 [file Image_1.tif]

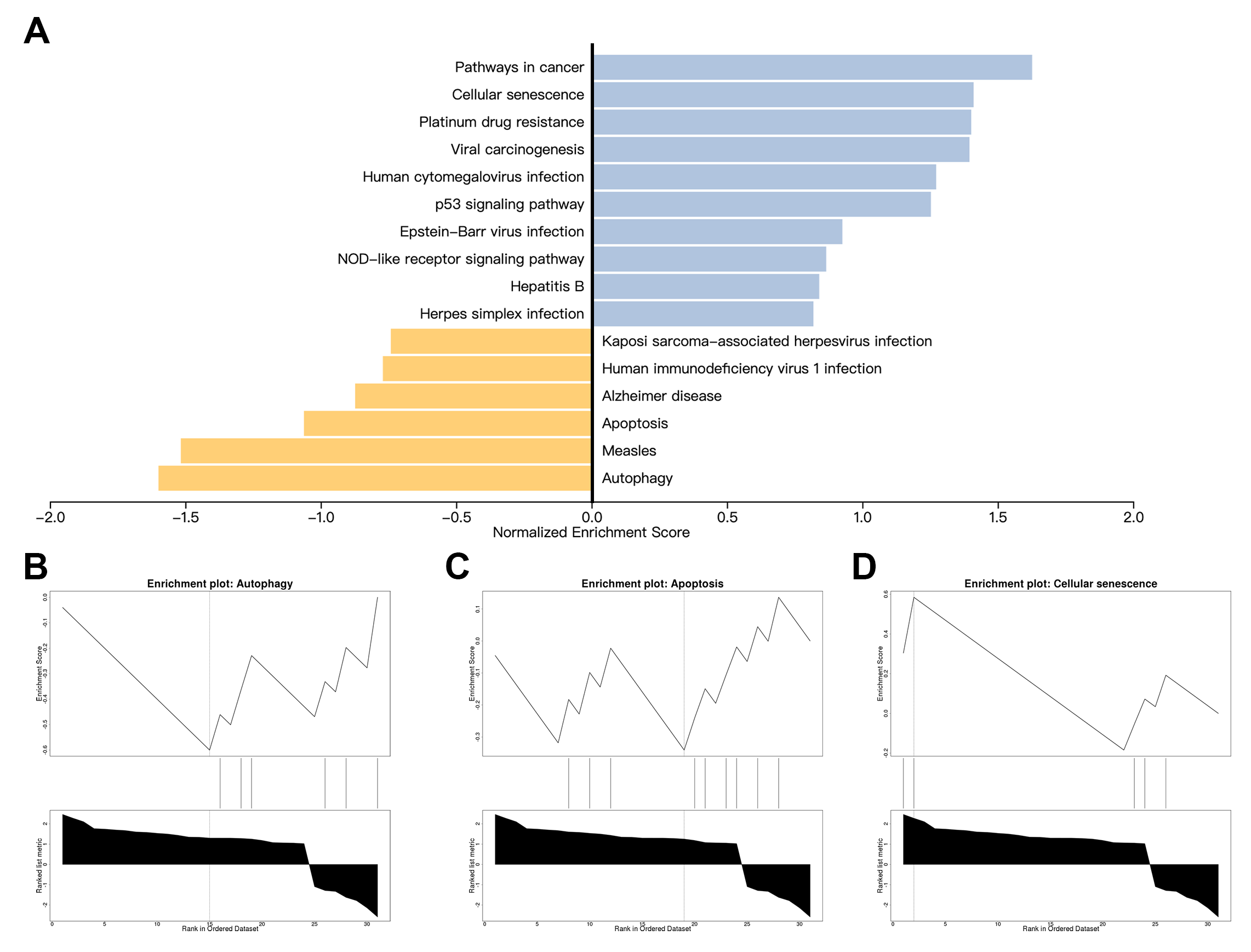

Supplement: Supplementary file 2 [file Image_2.tif]

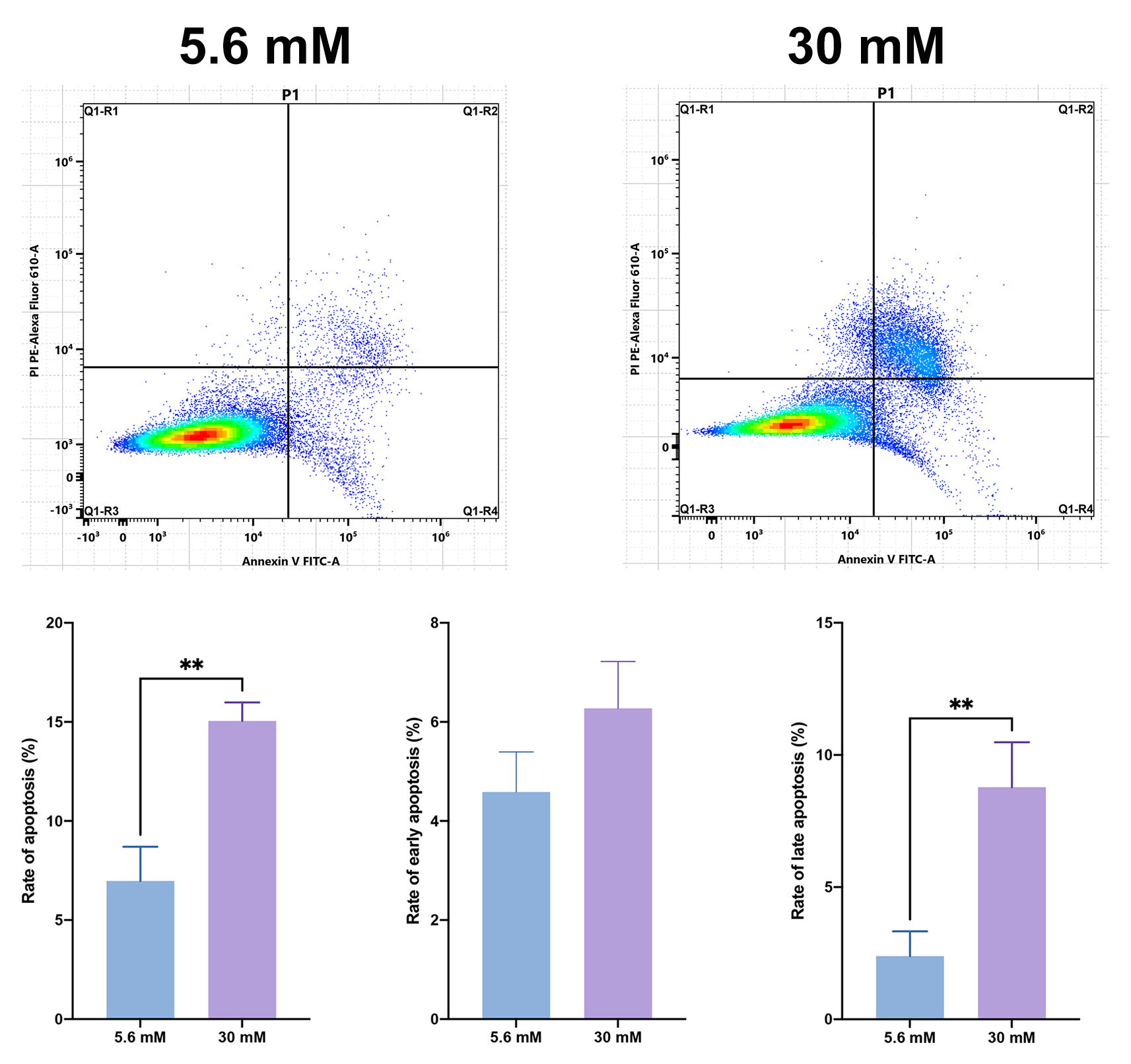

Supplement: Supplementary file 3 [file Image_3.tif]
