# Supplementary material for: Autophagy and senescence of rat retinal precursor cells under high glucose
Source: Front Endocrinol (Lausanne). 2023 Jan 4;13:1047642. doi: 10.3389/fendo.2022.1047642 (PMC9846177; doi:10.3389/fendo.2022.1047642)
Supplement: Supplementary file 4 [file Table_1.docx]

**SUPPLEMENTARY FILES**

**TABLE S1** Primer Sequences of RT-qPCR.

| **Genes** | **Sequences (5’-3’)** |
| --- | --- |
| *TP53* | **Forward:** TCCGGTCAGTTGTTGGA  **Reverse:** GCAGAGTGGAGGAAATGG |
| *CASP3* | **Forward:** TGGACAACAACGAAACCTC  **Reverse:** ACACAAGCCCATTTCAGG |
| *CCL2* | **Forward:** GTG​TCC​CAA​AGA​AGC​TGT​AGT​ATT​T  **Reverse:** TGC​TGA​AGT​CCT​TAG​GGT​TGA​T |
| *HIFA* | **Forward:** TCCATTTTCAGCTCAGGACACT  **Reverse:** GGTAGGTTTCTGTAACTGGGTCT |
| *CASP1* | **Forward:** CGGGCAAGCCAGATGTTTAT  **Reverse:** AACCACTCGGTCCAGGAAATG |
| *CDKN1A* | **Forward:** GCTGTCTTGCACTCTGGTGTCTC |
|  | **Reverse:** TGATAGAAATCTGTTAGGCTGGTCTGC |
| *CDKN2A* | **Forward:** GAGGGCTTCCTAGACACTCTG |
|  | **Reverse:** CGCAAATACCGCACGAC |
| *β-actin* | **Forward:** TGTCACCAACTGGGACGATA  **Reverse:** GGGGTGTTGAAGGTCTCAAA |
